# Design and Characterization of a Hyperspectral Colposcope Based on Dual-LCTF VNIR Narrow-Band Illumination

**DOI:** 10.3390/s26041255

**Published:** 2026-02-14

**Authors:** Carlos Vega, Raquel Leon, Norberto Medina, Himar Fabelo, Alicia Martín, Gustavo M. Callico

**Affiliations:** 1 Institute for Applied Microelectronics (IUMA), University of Las Palmas de Gran Canaria (ULPGC), 35017 Las Palmas de Gran Canaria, Spain; slmartin@iuma.ulpgc.es (R.L.); hfabelo@iuma.ulpgc.es (H.F.); gustavo@iuma.ulpgc.es (G.M.C.); 2 Complejo Hospitalario Universitario Insular Materno Infantil (CHUIMI), Servicio Canario de Salud (SCS), 35016 Las Palmas de Gran Canaria, Spain; 3 Fundación Canaria Instituto de Investigación Sanitaria de Canarias (FIISC), 35019 Las Palmas de Gran Canaria, Spain; 4 Research Unit, Hospital Universitario de Gran Canaria Doctor Negrin, 35019 Las Palmas de Gran Canaria, Spain; 5 Instituto de Investigación Sanitaria de Canarias (IISC), 35019 Las Palmas de Gran Canaria, Spain

**Keywords:** hyperspectral imaging, liquid crystal tunable filter, cervical cancer, image processing, medical imaging, medical instrumentation, colposcopy

## Abstract

**Highlights:**

**What are the main findings?**

- A dual-LCTF hyperspectral colposcope with emission-filtering and wavelength-dependent exposure was developed and quantitatively characterized over an extended VNIR range (460–1000 nm).
- Spectral scanning using LCTF technology provided improved robustness to motion artefacts and reduced acquisition time compared with a Snapscan-based hyperspectral colposcope.

**What are the implications of the main finding?**

- Emission-filtering combined with wavelength-dependent exposure provides a clinically robust alternative to spatio-spectral scanning for in vivo hyperspectral applications.
- Improved acquisition robustness and reduced motion artefacts support the integration of hyperspectral imaging into real-world cervical cancer screening workflows.

**Abstract:**

Early detection of precancerous cervical lesions is critical for improving patient management and clinical outcomes. Hyperspectral imaging has emerged as a promising non-invasive, label-free imaging modality for rapid medical diagnosis. This work presents the development of a liquid-crystal-tunable-filter-based hyperspectral colposcopy system covering the visible and near-infrared spectral ranges. The proposed system integrates two tunable filters into an existing Optomic OP-C5 clinical colposcope, enabling hyperspectral acquisition from 460 to 1000 nm with 130 spectral bands at 5 nm resolution using a panchromatic camera. Two alternative acquisition strategies were investigated: (i) filtering the light received by the system, or (ii) filtering the light emitted toward the sample. In addition, wavelength-dependent exposure control was studied to compensate for reduced system sensitivity and improve the signal-to-noise ratio in low-efficiency spectral regions. The system was benchmarked against a previous custom hyperspectral implementation based on a commercial camera. The comparative analysis highlights the advantages and limitations of both approaches, demonstrating the proposed system’s suitability for integration into clinical workflows and its potential for early detection of precancerous cervical lesions during routine colposcopic examinations.

## 1. Introduction

In Europe, cervical cancer affects approximately 15 women per 100,000 inhabitants, although significant differences exist across regions [1]. Globally, cervical cancer is recognized as a major public health concern, ranking fourth in terms of incidence and third in mortality among women worldwide in 2020, and affecting younger populations under the age of 45 at an alarming rate [2]. These statistics emphasize the necessity for improved early detection and screening tools for cervical cancer [3]. Currently, this screening is performed by combining colposcopy and cervical biopsies. However, this conventional method is inherently subjective and depends on the practitioner’s expertise, leading to a notable variability in results [4]. Previous studies report low sensitivity (between 68.5% and 77%) and specificity (between 75.9% and 82%) using this method [5,6], often leading to unnecessary biopsies [7].

Hyperspectral (HS) imaging (HSI) has emerged as a promising non-invasive and marker-free modality capable of quantifying subtle spectral and physiological changes in biological tissues [8], especially for cancer analysis [9]. In the context of cervical pathology, several studies have demonstrated its potential to reduce examiner-dependent variability and improve lesion detection accuracy. In 2016, Wang et al. presented a preliminary study claiming that spectral analysis in the 600–800 nm range (particularly at 620, 696, and 772 nm) was sufficient to classify normal, inflammatory, and high-grade lesions using a monochrome camera attached to an acousto-optic tunable filter [10,11]. A recent work by Jurjut et al. [12] presented further evidence that HSI can objectively differentiate cervical intraepithelial neoplasia (CIN) lesions from healthy cervical tissue by revealing significant increases in hemoglobin and water content, reflecting the angiogenesis and stromal alterations associated with neoplastic progression. While these results highlight the diagnostic potential of HSI, the acquisition system used was not optimized for routine clinical colposcopy, relying on a fixed long working distance, manual cervical stabilization, and post hoc spatial registration between separate imaging systems. These limitations emphasize the need for dedicated HS colposcopy solutions that are specifically designed for close-range cervical imaging and seamless integration into clinical workflows [13].

There are several technologies available for HSI acquisition, and they are usually classified by how the HS cube is composed [14]. In this work, we focus on two technologies: (i) spatio-spectral scanning and (ii) spectral scanning. Spatio-spectral scanning technology works by the movement of the sensor inside the camera. At each instant, the sensor captures a diagonal plane of the HS cube, covering a region of the spatial area. The HS cube is created by capturing a series of frames, which are added diagonally to the cube with a spatial shift between them. An example of this technology is the Snapscan HS camera (IMEC, Leuven, Belgium) [15], which features a Gaussian filter aligned with each sensor column, each tuned to a specific wavelength, and has been extensively used in medical imaging applications [16,17,18,19]. In contrast, spectral scanning technology allows scanning across a wide spectral range, capturing the complete scene for each wavelength to finally generate the HS cube [20]. The spectral resolution of these systems is limited by the filter’s spectral bandwidth. In addition, this technology allows the use of conventional panchromatic cameras as sensors, being able to achieve a high spatial resolution [21].

Liquid crystal tunable filters (LCTFs) employ spectral scanning technology. The light is filtered through a series of switchable liquid crystal cells interleaved between polarizing elements [22]. Light first passes through a linear polarizer, letting only one polarization reach the filter. Inside the LCTF, layers of liquid crystals and polarizers work together to control the transmitted wavelength. Due to birefringence, light passing through the liquid crystal splits into two rays with different polarizations, allowing certain wavelengths to pass to the next stage depending on the applied voltage. Although adding more stages can reduce the filter’s bandwidth, it also decreases light transmission, lowering image quality and increasing acquisition time. Therefore, a balance is needed between spectral resolution, light efficiency, and acquisition speed [22].

An LCTF-based system has two main strategies for capturing HS information [23]: (i) filtering the light received by the system; or (ii) filtering the light emitted to the sample. In the first approach, and the most common, the LCTF is placed in the image path between the sample and the sensor. The sample is illuminated with broadband light, and the filter sequentially transmits one spectral band at a time toward the sensor [24,25]. In the second approach, the filter is located in the illumination path, producing a narrow-band illumination system in which only a selected wavelength reaches the sample in each acquisition step [20]. Both strategies enable wavelength-selective imaging; the placement of the LCTF leads to different trade-offs in optical throughput, system complexity, and how the light is delivered to and collected from the sample [26].

This work focuses on the development of LCTF-based HS colposcope systems for the cervical examination covering the visible and near-infrared (VNIR) spectral range. This development was based on different stages. First, two strategies for capturing HS data were evaluated. Later, wavelength-dependent exposure control is studied to compensate for reduced system sensitivity and improve the signal-to-noise ratio in low-efficiency spectral regions. Finally, the performance of the LCTF-based HS colposcope was compared with that of a Snapscan-based colposcope, and the limitations and improvements of each system were analyzed. The development of a new system based on dual-LCTF VNIR narrow-band illumination aims to overcome the limitations presented and facilitate the integration of HSI into existing optical platforms.

## 2. Materials and Methods

**Figure 1:**
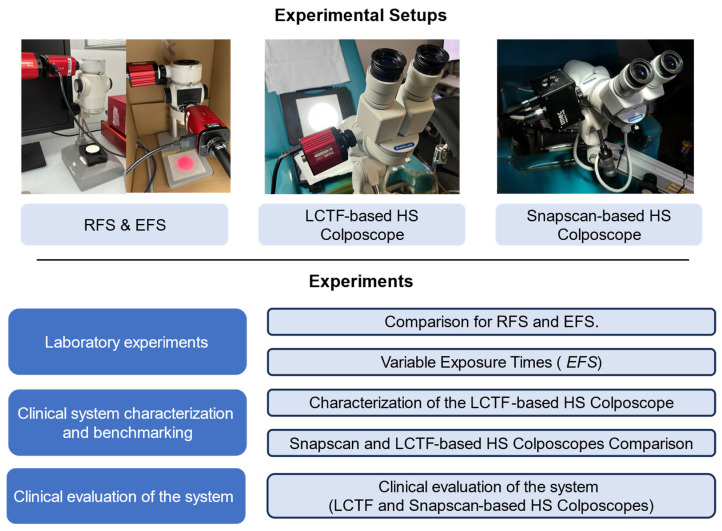
Summary of the experimental setups and experiments.

This section describes the experimental setups employed in this work (Figure 1) in which three different HSI setups were implemented. The first setup was designed to evaluate two different light-filtering strategies using LCTF. After selecting the best filtering approach, the second configuration applied this method to a commercial colposcope. Finally, the third setup involved the same commercial colposcope integrated with a commercially available HS camera. This configuration served as a benchmark, allowing a performance comparison between the custom LCTF-based colposcope system and the commercial HS camera. These experimental setups were employed in the different experiments to evaluate acquisition strategies under laboratory conditions, characterize and benchmark the clinical systems, and analyze cervix spectral properties in real clinical scenarios.

### 2.1. Reception-Filtering and Emission-Filtering Setups

In this study, two acquisition strategies were implemented using a common optical platform: a reception-filtering setup (RFS) and an emission-filtering setup (EFS). Both systems employ a single Kurios-XE2 tunable filter (Thorlabs, Inc., Newton, NJ, USA). This filter is designed to operate in the near-infrared (NIR) range, operating in the 650–1100 nm range that was divided into 90 spectral bands of 5 nm. Data are acquired by a CS135MUN (Thorlabs, Inc., Newton, NJ, USA) panchromatic camera, which has a high spatial resolution (1280 × 1024 pixels) and 10-bit resolution. This specific model presents enhanced performance in the NIR range, with a quantum efficiency peaking at 60% around 600 nm and gradually decreasing to 5% at 1000 nm. Additionally, a broadband halogen light source was selected due to its constant emission spectrum spanning 400–1750 nm. Specifically, the OSL2IR (Thorlabs, Inc., Newton, NJ, USA) was used, which includes a bulb with an aluminum-coated reflector for enhanced infrared (IR) performance. The OSL2IR is designed to channel the emitted light through a fiber optic guide to subsequent optical components.

**Figure 2:**
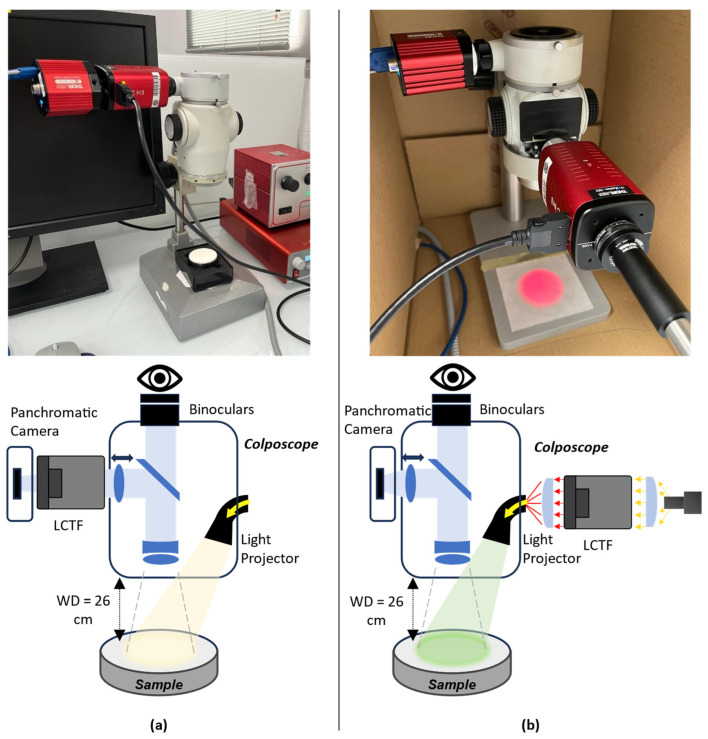
Representation of the different configurations of the system: (**a**) reception filtering setup and (**b**) emission filtering setup.

In the RFS configuration (Figure 2a), the light is reflected from the sample, filtered by the LCTF, and received by the sensor. In this configuration, the illumination system and the colposcope head are connected using a fiber guide. Broadband illumination is delivered through a fiber-coupled light source to the illuminator prism, and the reflected light is collected through the imaging optics. Although the colposcope provides two channels for stereoscopic vision, only one channel was dedicated to HS image acquisition. A beamsplitter was employed to divide the received light between the binocular and the camera attached to the LCTF. In addition, a splitter optimized for a large focusing distance (100 mm) was used in this setup, enabling the integration of the LCTF between the camera and the colposcope body.

The EFS configuration filters the illumination before it reaches the sample (Figure 2b). The illumination system’s light is directed into the LCTF. A concave lens is attached to the end of the fiber to collimate the light rays inside the filter. A second converging lens is positioned at the output of the filter to concentrate the light onto a prism in the colposcope head, which then projects the light onto the sample.

Using these principles, the exposure time for each wavelength was independently adjusted to reach a target digital value that ensured optimal sensitivity while avoiding saturation. A maximum exposure limit was imposed in low-sensitivity regions to constrain acquisition time, at the cost of reduced signal-to-noise ratio (SNR) in those bands. This adaptive configuration enables balanced spectral performance and practical total acquisition durations for clinical imaging.

### 2.2. LCTF-Based HS Colposcope

**Figure 3:**
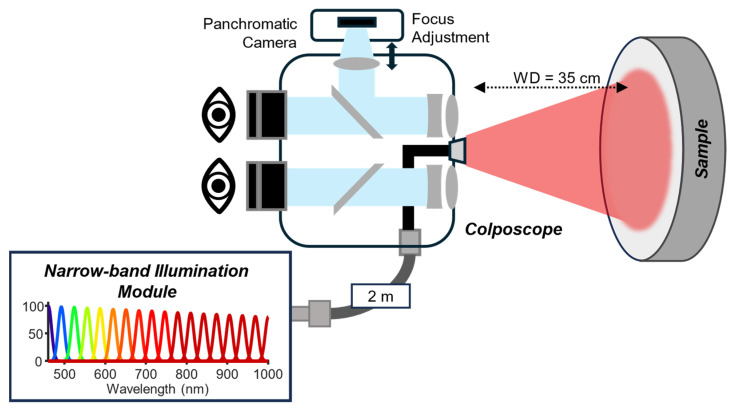
Representation of LCTF-based HS Colposcope.

Based on the results of the RFS and EFS comparisons, a clinical acquisition system was developed. The HS colposcope (Figure 3) is based on the OP-C5 colposcope (OPTOMIC España S.A, Madrid, Spain) with an external narrow-band illumination module (NBIM), and both are connected by a fiber guide. This NBIM incorporates two LCTFs (Kurios-VB1 and Kurios-XE2), which together provide nominal spectral coverage from 420 to 1100 nm, as defined by the manufacturers’ specifications. However, due to reduced illumination power and detector sensitivity at the spectral extremes, particularly below 460 nm and above 1000 nm, the effective operating range of the complete system is 460–1000 nm. This range is used consistently throughout the experimental evaluations and clinical acquisitions presented in this work.

The Kurios-VB1 filter covers the visible part and is illuminated by a high-CRI (>90%) LED source that produces a uniform spectrum from 420 to 700 nm, whereas the Kurios-XE2 filter covers the NIR part and is illuminated by a halogen source covering 500–1600 nm. In both channels, the emitted light is collimated before entering the filters to ensure stable and efficient spectral transmission. In addition, a white light source was integrated for visualization. A motorized mirror selectively directs each light path toward the output coupling optics. The mirror is digitally controlled by an Arduino Uno (Arduino, Monza, Italy) and can rotate between 0° and 190° to align the collimated beam with the output port. To use the visible filter, the mirror remains at 0°, while for the NIR filter, it rotates to 45° to redirect the beam positioned orthogonally to the output. The white light source requires a 135° orientation to reach the same output path. A converging lens then focuses the selected beam into the fiber guide for delivery to the colposcope. Finally, the image is acquired by a CS126MU (Thorlabs, Inc., NJ, USA), which offers a resolution of 12.3 MP, a high efficiency response (72% over 525–580 nm), 12-bit resolution, and reduced read noise (<2.5 e- RMS). Due to limitations in the optical field of view, only a central region of the sensor is effectively used, resulting in an effective spatial resolution of 1600 × 1600 pixels (2.56 MP).

### 2.3. Snapscan-Based HS Colposcope

The Snapscan-based HS colposcope [27] was initially designed to incorporate a commercial HS camera (Snapscan VNIR, IMEC, Leuven, Belgium) into an existing commercial colposcope (OP-C5, OPTOMIC España S.A, Spain). The system was able to capture 150 bands, covering a spectral range between 470 and 900 nm. However, after the integration with the colposcope, the system presented several limitations. The long acquisition time led to motion artefacts during clinical procedures. Furthermore, the sensor had limited spectral sensitivity above 900 nm, which restricted detection in the NIR spectral range. In addition, the sensitivity of the system varied significantly across different spectral bands, a characteristic inherent to the sensor, which caused underexposure in certain bands.

## 3. Experiments

To evaluate the performance, reliability, and clinical applicability of the proposed HS colposcope, a series of laboratory and clinical experiments were conducted (Figure 1). The experiments were divided into three groups:

(i). Laboratory experiments to assess the influence of the different LCTF configurations and determine the acquisition parameters required to maximize spectral performance.
(ii). Clinical system characterization and benchmarking to characterize the commercial colposcope using the LCTF-based system and to compare its performance with respect to a Snapscan-based HS colposcope previously developed by our group.
(iii). Clinical evaluation to assess the practical feasibility of the system during routine cervical examinations.

### 3.1. Laboratory Experiments

The first set of experiments was carried out under controlled laboratory conditions to characterize the behavior of the LCTF using the proposed setups and to evaluate the acquisition settings required to maximize signal quality across the spectral range. These tests provided a stable and reproducible environment to assess the impact of filtering mode, illumination conditions, and exposure strategy on the system’s overall performance.

#### 3.1.1. Comparison of RFS and EFS

The first experiment evaluated two LCTF integration strategies: RFS and EFS. For both configurations, the imaging system was optimized by adjusting focus and field of view on a USAF spatial target (Edmund Optics, Barrington, NJ, USA). Although the optical pathways were kept as similar as possible, the introduction of a 100 mm working-distance beamsplitter in the RFS configuration resulted in a slightly reduced field of view.

Spectral accuracy was assessed using the Zenith Polymer SG3333 Standard (SphereOptics, Herrsching, Germany), which contains rare-earth absorbers with well-defined spectral features. For each setup, an HS cube was acquired under the same illumination conditions, and the mean spectral signatures were extracted from a uniform region of the reference material. The root-mean-square error (RMSE) between the measured spectrum and the manufacturer-provided reference spectrum was calculated according to Equation (1):

(1) $$RMSE=\sqrt{\frac{\sum^{N}{\left(y_{i}-{\hat{y}}_{i}\right)}^{2}}{N}},$$

where $y_{i}$ is the reference reflectance, ${\hat{y}}_{i}$ is the measured reflectance at each spectral band (i), and N is the total number of discrete spectral bands (wavelength samples) within the evaluated spectral range. This metric quantified how accurately each configuration reproduced the expected spectral response.

#### 3.1.2. Variable Exposure Times

The second experiment evaluated the impact of adaptive exposure settings on spectral performance. Using the EFS configuration, the system efficiency was characterized over 650–1090 nm, and wavelength-dependent exposure times were calculated using the proposed method. The Zenith Polymer was then captured to assess the spectral performance. Three exposure strategies were compared:

(1) Constant, using the same exposure time for all bands;
(2) Short-limit, with a maximum of 500 ms per band;
(3) Long-limit, with a maximum of 1200 ms per band.

Characterization measurements were acquired every 20 nm to reduce experimental duration. The final exposure profile, matching the 5 nm spectral resolution used during the HS image acquisition, was generated by spline interpolation of the intermediate points.

### 3.2. Clinical System Characterization and Benchmarking

After establishing the system’s behavior under controlled laboratory conditions and selecting the optimal configuration and exposure strategy, the next set of experiments was focused on validating its performance in the clinical environment. Furthermore, its performance was benchmarked against our previous system, based on a Snapscan HS camera, which exhibited several limitations [27]. This comparison allows us to determine whether the newly developed system provides measurable improvements in acquisition quality and its suitability for clinical practice.

#### 3.2.1. Characterization of the LCTF-Based HS Colposcope

Before clinical application, the LCTF-based HS colposcope was characterized to validate its performance. Initially, the system efficiency was characterized over the 460–1000 nm spectral range, and wavelength-dependent exposure times were calculated according to the presented method. For this experiment, the objective value for the exposure time was set to 3000 digital numbers (DN), given the 12-bit resolution of the CS126MU camera (Thorlabs Inc., Newton, NJ, USA), and the maximum exposure time is set to 300 ms. The Zenith Polymer was captured under clinical conditions with a low-light environment (below 3.0 Lux), and the resulting spectral signatures were compared with the manufacturer’s reference data to compute RMSE.

Spectral linearity of the system was assessed by using the Rez Checker target (Edmund Optics, NJ, USA), which includes a 6 × 7 matrix of calibrated color patches and a central grayscale. The spectra from the grey-scale tiles are ordered from 1 to 12, corresponding to 95% to 24% reflectivity, respectively. For each tile, the average spectrum was calculated from a 10 × 10 pixel region at the center.

Finally, the overall electro-optical transfer characteristics were analyzed in accordance with ISO 14524:2009 [28]. The opto-electronic conversion function (OECF) was analyzed by plotting the status T density against the mean measured reflectance of the grayscale tiles for all the bands. The standard defines that, under ideal conditions, a straight line is represented between values, while any error in the quantification process results in a deviation from this reference.

#### 3.2.2. Snapscan- and LCTF-Based HS Colposcope Comparison

The performance of the LCTF-based system was benchmarked against a state-of-the-art Snapscan-based HS colposcope [27], focusing on acquisition speed, spectral coverage, spatial resolution, and signal efficiency. This is a system-level comparison aimed at evaluating clinical practicality, not a strict hardware fairness test. Both systems were evaluated under comparable clinical illumination conditions (below 3.0 lux).

Spectral performance was first assessed by capturing the Zenith Polymer to analyze spectral fidelity and resolution. Subsequently, spectral linearity was evaluated using the Rez Checker target, as described in the previous experiment. The dynamic range (DR) was computed to quantify the system’s ability to discriminate between the highest and lowest measurable signal levels. For reflectance-based measurements, the maximum signal corresponds to complete light reflection, whereas the minimum corresponds to minimal reflected light. The DR is computed using Equation (2), where $W_{r}$ and $D_{r}$ are extracted from the mean digital value in the 95% reflectance element and from the mean digital value in the 20% reflectance element in the Rez Checker, respectively.

(2) $$DR(db)=20\log\left(\frac{mean(W_{r})}{\begin{array}{c} mean(D_{r}) \end{array}}\;\right)$$

The SNR was also computed to evaluate the sensitivity and noise performance of the system. It is defined as the ratio between the average signal of the HS image ($HS_{data}$) and the dark current (DC) noise (Equation (3)). A high SNR indicates that the system can effectively distinguish the signal from the noise across all captured bands. For our system the maximum achievable SNR is measured by the mean digital value per wavelength in the 95% reflectance element in the Rez Checker and quantifying the dark current noise with the standard deviation (SD) in the 20% reflectance element.

(3) $$SNR(db)=20\log\left(\frac{mean\left(HS_{data}\right)}{SD(DC)}\right)$$

In this analysis, the SNR and DR are first computed independently for each wavelength across the full spectral range using the Rez Checker target. A global value is subsequently reported by averaging the per-band results over all 130 spectral bands.

### 3.3. Clinical Evaluation of the System

The LCTF-based HS colposcope system was clinically evaluated using in vivo cervical images acquired during routine gynecological screening. The dataset included three HS images from three different patients obtained on the same clinical day. For comparison, three additional patients acquired using the Snapscan-based HS colposcope were selected from the HyCervix dataset [29]. Each examination was conducted by a qualified gynecologist, who collected the patients’ demographic and clinical information and captured HS images of the cervix using the developed system.

To perform this comparison, three aspects were analyzed. First, the acquisition time was evaluated to assess the proposed system’s compatibility with routine clinical practice. Second, a qualitative visual inspection of the acquired HS cubes was conducted to evaluate spatial coherence, inter-band alignment, and the presence of motion-related artefacts. Finally, the spectral performance of both systems was compared by analyzing representative in vivo spectral signatures from the two main cervical tissue types: the exocervix and the endocervix.

All procedures followed current clinical and ethical standards and were approved by the ethical committee of the Complejo Hospitalario Universitario Insular-Materno Infantil (CHUIMI) with reference 2022-081-1. All the participants involved in this study and/or their legal guardians were informed about the research and voluntarily signed an informed consent form allowing their participation and the anonymous publication of the results. In addition, all research methodologies were performed in accordance with the current guidelines and regulations.

## 4. Experimental Results

### 4.1. Laboratory Experiments Results

#### 4.1.1. Comparison of RFS and EFS Results

**Figure 4:**
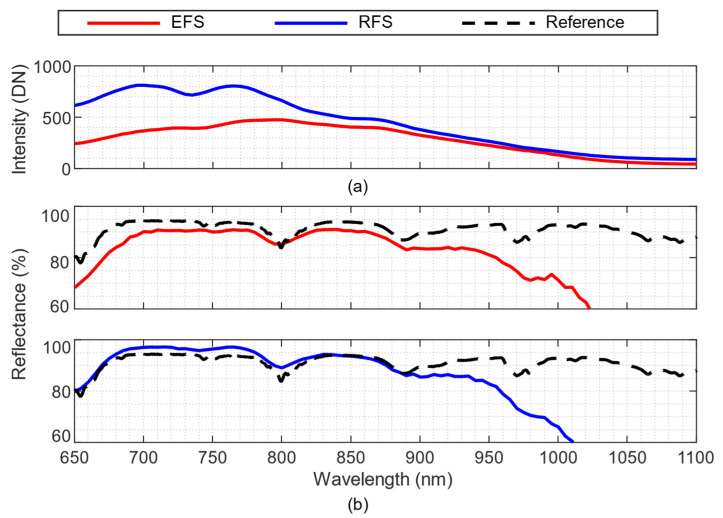
Comparison between the emission filtering setup and the reception filtering setup. (**a**) Represents the raw white reference, and (**b**) represents the calibrated spectral signature of the Zenith Polymer captured by both setups (red and blue) and the reference provided by the manufacturer (black). WR: white reference; EFS: emission filtering setup; RFS: reception filtering setup.

The first experiment compared the two LCTF integration strategies: RFS and EFS. Although both used the same optics, the RFS received 87.5% less light than the EFS, mainly due to the difference in the focal distance introduced by the 100 mm length of the LCTF in the RFS. To ensure a fair comparison between the configurations, the exposure times were independently adjusted to ensure that each system achieved an acceptable spectral response up to approximately 950 nm. Based on this criterion, the EFS configuration was operated with an exposure time of 250 ms, whereas the RFS required a substantially longer exposure time of 2000 ms. Figure 4a shows the raw white-reference spectral signatures after adjusting the exposure times. It can be observed that, in both configurations, the spectral responses decrease rapidly beyond 900 nm, suggesting a high potential for improved dynamic range with longer exposure times. This low response is mainly due to the sensor’s reduced quantum efficiency in the NIR region.

Figure 4b presents the calibrated spectral signatures from the Zenith Polymer. Both configurations matched the manufacturer’s reference curve up to ~880 nm, though high-frequency peaks were smoothed due to the 9–24 nm filter bandwidth. Beyond 880 nm, signal degradation was due to reduced sensor quantum efficiency. Differences were observed in the 675–730 nm range, where the RFS exhibited a slightly higher reflectance peak in the raw measurements; however, these differences were effectively compensated after calibration and did not affect the overall spectral agreement. Quantitatively, the EFS achieved an RMSE of 27.1%, compared to 29.0% for RFS. Given its higher efficiency, wider field of view, and reduced dimensions in the colposcope’s head unit, subsequent experiments were conducted using the EFS configuration.

#### 4.1.2. Variable Exposure Times Results

**Figure 5:**
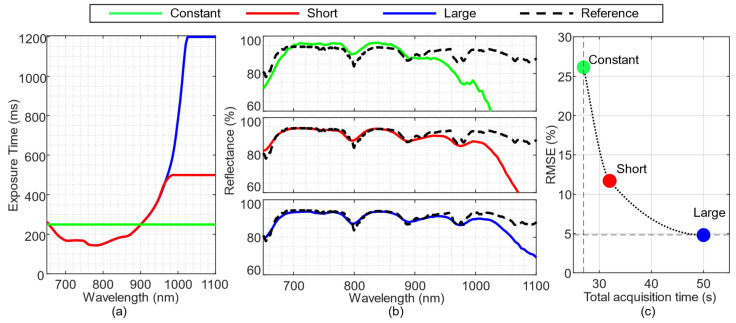
Comparison of the different exposure time configurations. (**a**) Exposure time calculated for each wavelength with different configurations: constant (green), short (red), and large (blue). (**b**) Zenith Polymer spectral signatures captured using each configuration and compared with the reference provided by the manufacturer (black). (**c**) Relationship between the RMSE of the captured spectra and the total HS acquisition time for each tested configuration. The dotted curve represents the trend estimated using a Piecewise Cubic Hermite Interpolating Polynomial (PCHIP).

The impact of variable exposure time was evaluated using the EFS. Initially, the acquisition time was characterized, and the wavelength-dependent exposure times were calculated according to the method presented in [30]. The resulting exposure times are shown in Figure 5a using three different configurations (short, large, and constant): (i) a variable exposure with a short maximum (500 ms), (ii) a variable exposure with a long maximum (1200 ms), and (iii) a constant exposure of 250 ms used as a reference. Both configurations employing the variable-exposure method exhibited similar exposure values up to 970 nm. Beyond this wavelength, the short configuration reached its maximum limit, whereas the long configuration extended the system sensitivity up to 1030 nm.

In order to analyze the behavior of the different exposure configurations, the Zenith Polymer was acquired, and the spectral signatures were compared in Figure 5b. Using the large configuration (blue line), the system was able to acquire the spectral signature with the highest precision (RMSE=5%) and keep a continuous acquisition value up to 1040 nm, where the reflectance value starts decreasing down to 80%. Compared to the baseline experiment (green line), the error for the constant configuration was higher (RMSE=26%), and with an increasing error from the reference curve over 880 nm. The short configuration (red line) exhibits intermediate performance between the two systems, achieving an RMSE of 12% while maintaining continuous acquisition up to 1010 nm.

Increasing the maximum exposure time introduces a trade-off between total acquisition time and the fidelity of the recovered spectral signatures. Figure 5c shows the relationship between RMSE and the total acquisition time of the HS cube. The constant configuration achieved an acquisition time of 28 s. In contrast, the short configuration required 32 s while reducing the RMSE by 54% (from 26% to 12%). The large configuration further reduced the RMSE by 81% (from 26% to 5%), at the expense of a substantially longer acquisition time of 50 s. When normalizing the RMSE reduction by the additional acquisition time, the short configuration achieved an average RMSE reduction of approximately 13.5% per additional second, whereas the large configuration yielded only about 4.5% per additional second. Based on this analysis, the subsequent experiments adopt a variable exposure-time strategy similar to the short configuration, with the maximum exposure time constrained to approximately twice that required under constant conditions, achieving a balanced compromise between spectral performance and practical acquisition time.

### 4.2. Clinical System Characterization and Benchmarking Results

#### 4.2.1. Characterization of the LCTF-Based HS Colposcope Results

The clinical acquisition system was developed based on the EFS configuration and extended the spectral range from 460 to 1000 nm using two LCTFs operating in parallel: the Kurios VB1 for the visible region and the Kurios XE2 for the NIR region. For all clinical characterization experiments, a wavelength-dependent variable exposure time was applied using a single configuration that was selected based on the results of the previous exposure analysis. Specifically, a variable exposure time profile was employed, adapted from the short configuration. The maximum exposure time was limited to 300 ms to balance spectral fidelity and total acquisition time.

**Figure 6:**
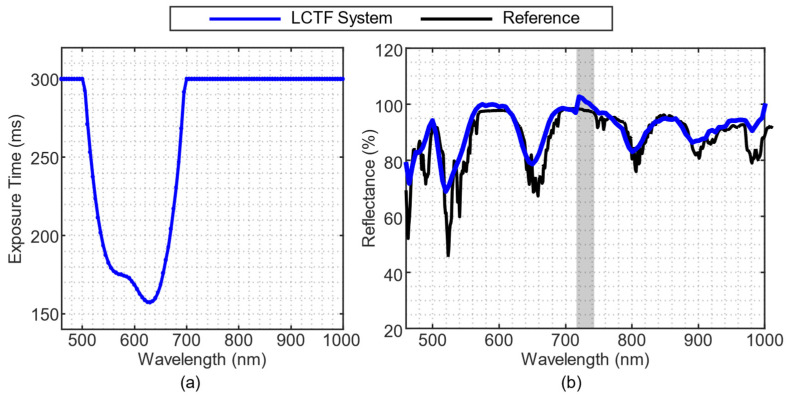
(**a**) Wavelength-dependent exposure time calculated for each spectral band using the adaptive exposure strategy. (**b**) Calibrated reflectance spectral signature of the polymer acquired with the LCTF-based HS colposcope (blue), compared with the manufacturer-provided reference (black). The spectral region affected by the transition between filters is highlighted in grey.

To validate the spectral response and acquisition times, the system was positioned facing a white reference under dark-room conditions. Figure 6a shows the exposure time computed for each band, where it can be observed that the most sensitive bands are between 500 and 600 nm with an overall acquisition time of 35 s. The calibrated reflectance spectrum of the Zenith Polymer is presented in Figure 6b. The measured signature closely matched the manufacturer’s reference, with an RMSE of 3.62%. A slight degradation in performance is observed beyond 950 nm, which is attributed to the reduced quantum efficiency of the sensor in the NIR region. In addition, a localized peak between 720 nm and 740 nm, marked by the gray-shaded region in Figure 6b, is present, corresponding to the transition between the filters. This artefact is caused by the rotation of the motorized mirror required to align the distinct optical paths of the two filters into the common emission fiber optic guide.

**Figure 7:**
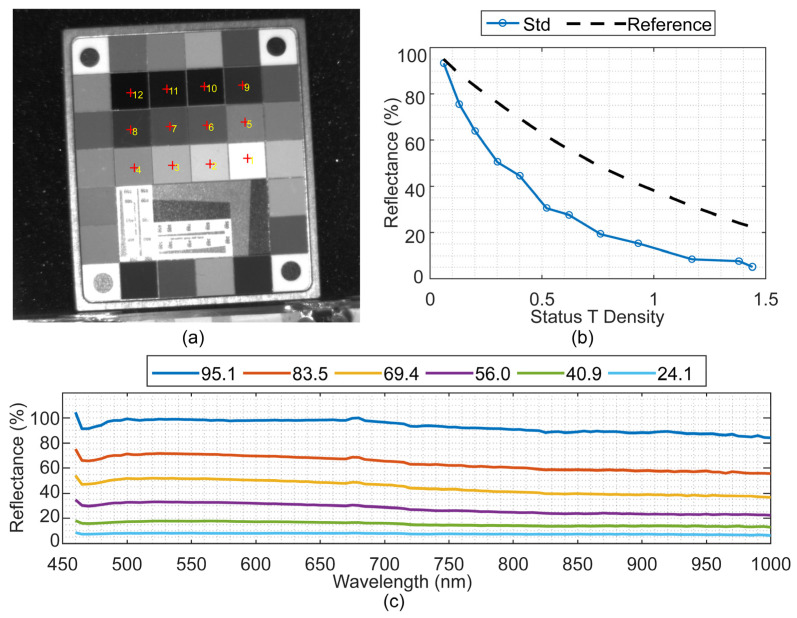
Radiometric and spectral linearity assessment of the LCTF-based HS colposcope using the Rez Checker target. (**a**) White-light image of the Rez Checker target showing the grayscale patches, ordered from high to low reflectance. (**b**) OECF profile for the LCTF system (blue) compared with the ideal linear response (dashed line). (**c**) Calibrated reflectance spectral signatures of the grayscale elements.

The Rez Checker (Figure 7a) was then captured to evaluate the system’s linearity to light intensity. Figure 7b shows the OECF profile, where the measured reflectance is plotted against the calibrated Status T density of the target. The LCTF-based system exhibits a near-linear response across the dynamic range, with greater deviations from the ideal reference line (dashed curve) at lower reflectance levels, where linearity becomes more pronounced. The reflectance spectral profiles of the grayscale elements are displayed in Figure 7c, confirming stable spectral performance and consistent separation between the reflectance levels across the 460–1000 nm spectral range. These results indicate that the LCTF-based HS colposcope provides a linear and reliable radiometric response suitable for quantitative reflectance measurements during clinical imaging.

#### 4.2.2. Snapscan- and LCTF-Based HS Colposcope Comparison Results

The spectral response and the dynamic range were compared between the proposed LCTF-based HS Colposcope (LCTF system) and the Snapscan-based HS Colposcope (Snapscan system). In terms of resolution, the LCTF system offers a higher effective spatial resolution of 1600 × 1600 pixels with an effective spectral range of 460–1000 nm, compared to the 900 × 1000 pixels and 470–900 nm range of the Snapscan system (Table 1). The latter offers a higher spectral resolution with 158 bands and an average full width at half maximum (FWHM) of 7 nm, compared to 109 bands and 18 nm FWHM of the LCTF system.

**Figure 8:**
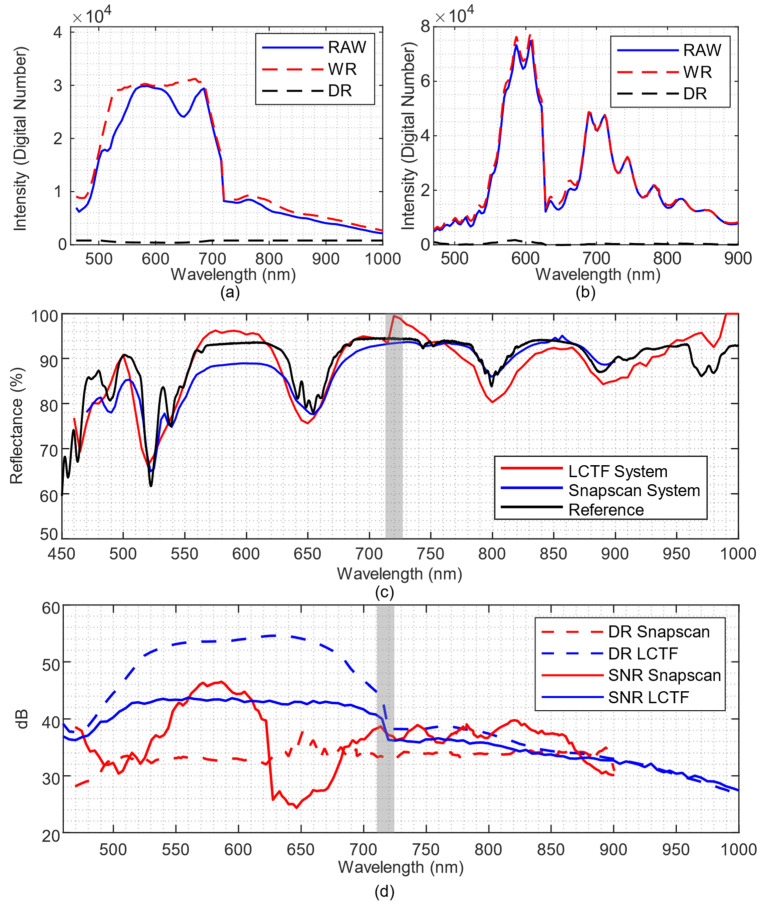
Comparison of the spectral performance of the proposed LCTF-based HS colposcope (LCTF) against the Snapscan-based HS colposcope (Snapscan). Raw image (blue), white reference (red), and dark reference (black) of (**a**) LCTF system and (**b**) Snapscan system. (**c**) Comparison of the Zenith Polymer spectral signatures captured with LCTF and Snapscan systems (red and blue, respectively) and the reference provided by the manufacturer (black). (**d**) DR and maximum SNR of both systems. The spectral region affected by the transition between the VNIR filters is highlighted in grey.

Figure 8a,b present the raw spectral signatures of the Zenith Polymer and the spectral signatures of the white and dark references for the LCTF system and the Snapscan system, respectively. The white reference extracted from the LCTF system (Figure 8a) shows two distinct regions: a stable, continuous one in the 520–700 nm spectral range, and a lower-amplitude region with a gradual decrease from 730 to 1000 nm. In contrast, the white reference captured by the Snapscan system (Figure 8b) shows a large intensity variation across bands, especially at the peaks around 600 and 700 nm. In terms of the dark reference, both systems present a stable value, which is lower for the Snapscan system with respect to the LCTF system (6 vs. 40 digital number, respectively). These results suggest that the proposed LCTF system provides a more consistent spectral performance with an almost constant sensitivity for wavelengths below 1030 nm.

Figure 8c shows the calibrated spectral signature of the Zenith Polymer acquired by both systems. Qualitative results show some differences between both systems, where the Snapscan system (blue) presents a lower reflectance value at shorter wavelengths (between 460–700 nm), while the LCTF system (red) introduces a larger error between 700–720 nm range, due to the transition between both LCTF filters. Quantitatively, results are similar across the common spectral range, with RMSEs of 3.14% for the Snapscan system and 3.62% for the LCTF system.

Figure 8d presents the maximum SNR and DR for each system and wavelength. The Snapscan system, operating at 10-bit resolution, exhibits a relatively stable DR of approximately 34 dB across the spectral range. However, its SNR shows marked variability, with pronounced drops in wavelength regions of lower sensitivity, particularly around 600–650 nm. In contrast, the LCTF system, which incorporates a 12-bit resolution sensor, achieves a higher DR in the visible range, peaking at approximately 55 dB between 520 and 650 nm, followed by a gradual decrease toward longer wavelengths due to reduced sensor efficiency in the NIR. Moreover, the LCTF system maintains a consistently high SNR of approximately 43 dB across most of the visible range, remaining above 32 dB beyond 900 nm. This demonstrates the effectiveness of the wavelength-dependent exposure time strategy in ensuring robust spectral quality.

### 4.3. Clinical Evaluation of the System Results

The performance of both LCTF-based and Snapscan-based HS colposcopes was evaluated during routine gynecological examinations at CHUIMI, with minimal disruption of the standard clinical workflow. The clinical assessment of the LCTF-based system was conducted to validate its performance, ensuring it is at least comparable to the reference Snapscan-based system. To this end, three patients were captured during a single clinical session and compared with an existing HyCervix dataset, collected with the Snapscan-based HS colposcope [28]. To perform this comparison, three aspects were analyzed: acquisition time, image quality, and spectral performance rather than diagnostic performance.

**Figure 9:**
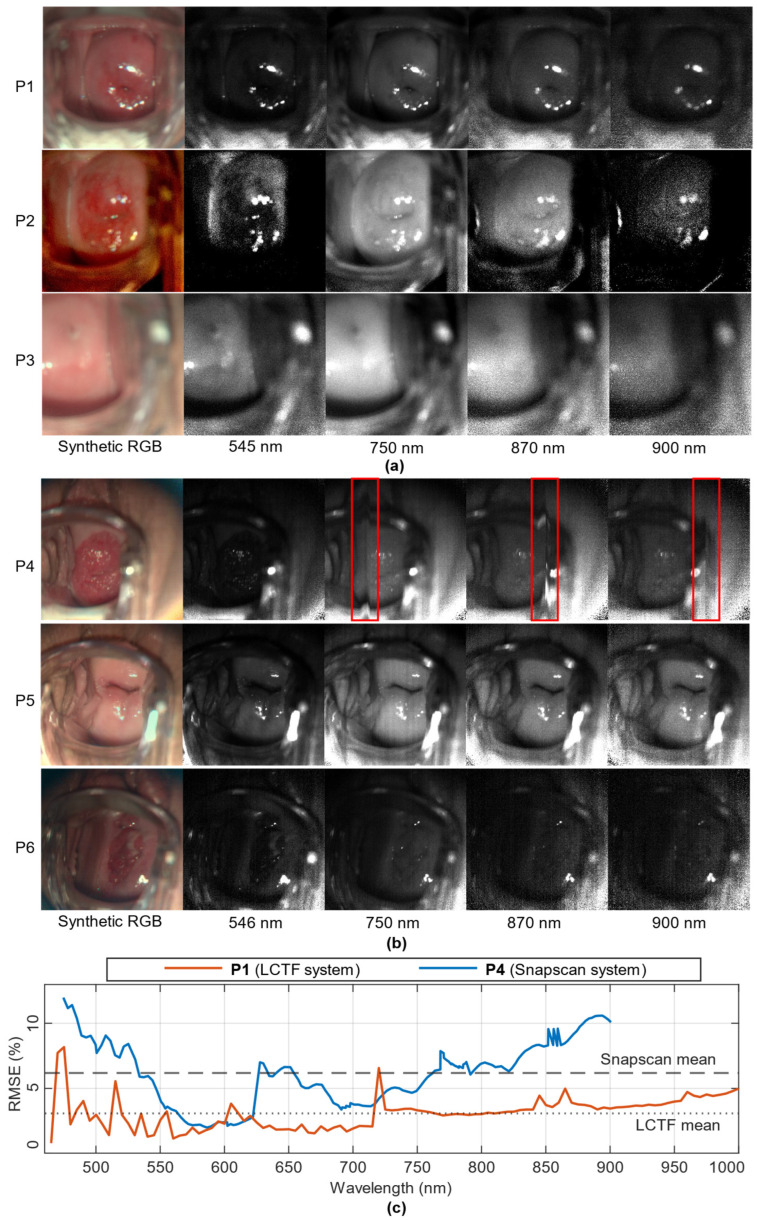
Representative in vivo HS cervical images acquired with the LCTF-based and Snapscan-based colposcopes. (**a**) In vivo HS cubes acquired with the LCTF-based system from three patients, visualized using a synthetic RGB composite and selected spectral bands at 545, 750, 850, and 900 nm. (**b**) HS cubes acquired with the Snapscan-based system from three representative patients, displayed using a synthetic RGB composite and selected spectral bands at 546, 750, 850, and 900 nm. Motion-induced vertical artefacts characteristic of spatio-spectral scanning are highlighted in red frame. (**c**) Inter-band RMSE computed between consecutive spectral bands for comparing patients P1 and P4 captured using the LCTF and Snapscan systems, respectively.

The acquisition time for each system was computed. The LCTF-based system required a mean of 35 s to acquire an HS cube, which is compatible with routine colposcopic practice and comparable to the 45 s reported for the Snapscan-based HS colposcope [28]. A qualitative visual inspection of the acquired HS cubes was performed to assess the presence of motion-related artefacts inherent to spatio-spectral scanning technology. These artefacts may be caused by either patient respiration or involuntary movements during the image acquisition, which can compromise the spatial and spectral integrity of the data. Figure 9a shows four representative wavelengths (545, 750, 870, and 900 nm) from the three HS cubes acquired with the LCTF-based system. High spatial coherence is observed, with no band misalignment or motion-induced distortions across all spectral bands. However, slight defocus due to operator error could be observed in P3, without affecting inter-band spatial consistency. In contrast, HS cubes acquired using the Snapscan system (Figure 9b) exhibited, in P4, motion-induced vertical acquisition artefacts resulting from the spatio-spectral scanning process of the HS camera and from involuntary patient motion during acquisition. Furthermore, P5 exhibits a distinctive noise pattern along the horizontal axis, which is especially noticeable at 750 nm. The motion-induced artefacts were further analyzed in Figure 9c by computing the inter-band RMSE, defined as the error between each spectral band and its immediately preceding band. Results are presented for patient P1 acquired with the LCTF system (orange) and patient P4 acquired with the Snapscan (blue) system. P4, which is affected by motion artefacts, exhibits a systematically higher inter-band error than P1. This increase is particularly pronounced beyond 750 nm, where artefacts are also evident in the spectral error curves. When averaged across all bands, the mean RMSE for P4 is 6.2%, compared to 3.1% for P1. These examples demonstrate how motion artefacts in the Snapscan system, inherent to spatio-spectral scanning technology, introduce inconsistencies between bands, potentially affecting the reliability of spectral signatures.

**Figure 10:**
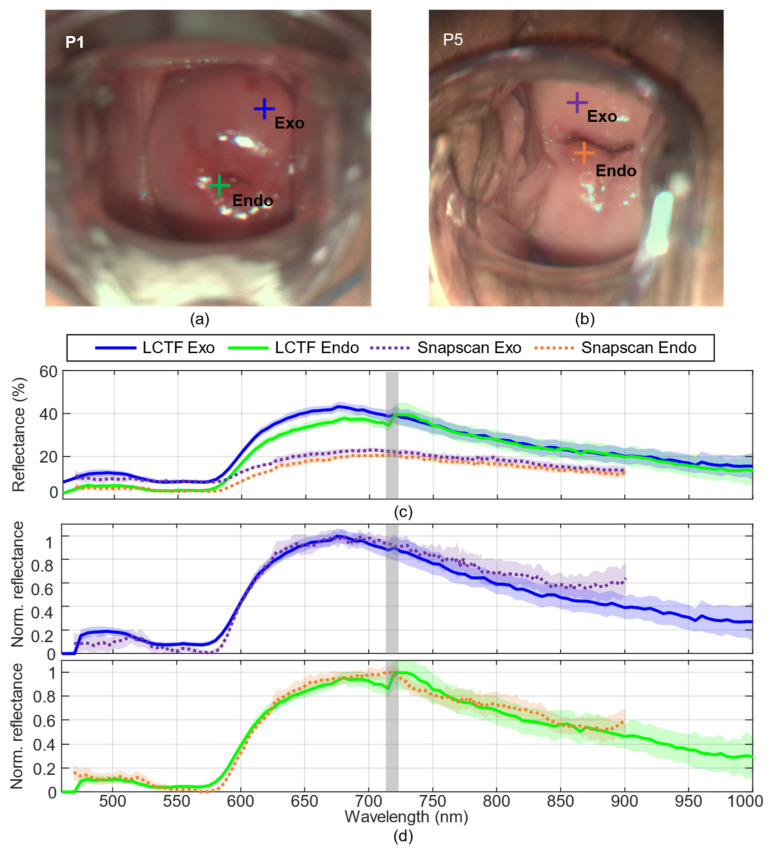
Clinical comparison of exocervical and endocervical spectral signatures acquired with the LCTF and Snapscan systems. (**a**) In vivo synthetic RGB image from patient 1 (P1) captured with the LCTF system showing selected representative regions of interest. (**b**) In vivo synthetic RGB image from patient 5 (P5) captured with the Snapscan system showing the corresponding representative regions of interest. (**c**) Representative reflectance spectral signatures extracted from the exocervix and endocervix of each patient. (**d**) The same spectral signatures after min–max normalization, with exocervical and endocervical tissues plotted independently. The spectral region affected by the transition between the VNIR filters is highlighted in grey.

In addition, a comparative spectral analysis of the main cervical tissue types was conducted using two representative patients (P1 and P5), both of whom were diagnosed as HPV-positive without evidence of cervical lesions. For each patient, two regions of interest were selected independently: one corresponding to the exocervix (referred to Exo in Figure 10) and one to the endocervix (referred to Endo in Figure 10), as indicated in the RGB images (Figure 10a,b).

Figure 10c presents the reflectance spectra acquired using the LCTF-based and Snapscan-based colposcopes. Each spectral signature corresponds to the mean reflectance from a 5 × 5-pixel region of interest, with the associated standard deviation shown in the background. Spectra acquired with the LCTF-based system (blue and green) exhibit smooth, continuous spectra across the 460–1000 nm range. A minor discontinuity is observed in the 700–750 nm range due to the transition between both LCTFs, as analyzed in Section 4.2. In contrast, the Snapscan-based spectra (dotted lines) show a lower overall reflectance level, likely due to differences in illumination projection, cervix geometry, and tissue depth.

For improved comparison, Figure 10d shows the same spectral signatures after applying min–max normalization [31]. The normalized exocervix spectral signature exhibits a similar trend across both systems, with minor differences in the depth of the reflectance valley around 540–580 nm. In endocervical tissue, larger differences are observed in the 700–750 nm range, once more associated with the LCTF transition region. In both tissues, the LCTF system provides a continuous and stable normalized response beyond 900 nm, whereas the Snapscan system is limited to the 470–900 nm range and displays increased band-to-band fluctuations and slightly higher variability. Globally, these results highlight the LCTF system’s improved spectral stability compared with the Snapscan system. In addition, the LCTF system captures a broader wavelength range, providing additional information that may assist in differentiating tissues within this spectral region.

## 5. Discussion and Conclusions

This research presents the development and subsequent evaluation of an LCTF-based HS colposcope, targeting the enhanced detection and classification of cervical neoplasia during routine medical examinations. This system was designed to overcome the limitations of the Snapscan-based HS colposcope developed in a previous study, which has been used to collect data, as a proof-of-concept system, during clinical routine practice for over a year. This work presents an extensive characterization and evaluation of LCTF technology for acquiring HS images with high spectral response in the NIR range, and its specific application in colposcopy.

Initially, two approaches were evaluated for integrating LCTF into the light path. The EFS configuration filters the light that is emitted to the sample, while the RFS configuration filters the light received by the sensor, which is the predominant approach in the literature. The comparison concluded that both configurations were capable of capturing spectral signatures up to 900 nm with high precision. However, EFS was favored for practical clinical application due to its convenience, extended field of view, and reduced dimensions on the colposcope’s head unit.

A method of variable exposure time for each wavelength was introduced into the EFS to enhance the low-sensitivity regions. Results showed significant improvements in the spectral response and uniformity of the system’s output. By adjusting exposure times to optimize the SNR across all bands, a more consistent spectral response was achieved, mitigating the variability inherent to different wavelengths and intensities. This method was subsequently applied to the LCTF-based HS colposcope using a strategy similar to the short configuration, limiting the maximum exposure time to approximately twice that required under constant conditions to achieve a balance between spectral performance and practical acquisition time.

Improvements over the system developed in the previous work were distinctly evident. The Snapscan-based HS colposcope system exhibited significant sensitivity fluctuations across different wavelengths, which negatively affected SNR in some specific bands, especially in the 630–680 nm range. In contrast, the LCTF-based HS colposcope exhibited higher DR, peaking at approximately 55 dB, and a more uniform SNR, further validating the effectiveness of the wavelength-dependent exposure strategy in ensuring robust spectral quality. Also, the LCTF system presented an extended acquisition range of 460–1000 nm instead of 470–900 nm from the Snapscan system, while maintaining high precision in the common spectral range (RMSE = 3.62%). The extension beyond 900 nm is particularly relevant in light of previous findings by Jurjut et al. [12], who reported significant increases in hemoglobin and water content in CIN lesions, with the water content index being strongly influenced by absorption features present from approximately 940 nm onward [32].

A preliminary clinical evaluation of the LCTF system was performed on three different patients as a proof-of-feasibility study, with the objective of assessing clinical usability and acquisition robustness rather than diagnostic performance. In these initial acquisitions, the HS cubes exhibited high spatial coherence and a smooth spectral response. Compared with the Snapscan-based HS colposcope, the proposed LCTF system did not exhibit motion-induced vertical acquisition artefacts due to patient involuntary movements and reduced acquisition time from 45 to 35 s. In addition, the comparative analysis of exocervical and endocervical tissues from two patients showed similar spectral trends in both systems, with minor differences observed mainly in the 700–750 nm range due to the transition between the two LCTFs. Furthermore, the LCTF system exhibited reduced inter-band fluctuations and more stable spectral signatures, particularly beyond 900 nm, compared with the Snapscan system.

Overall, the presented LCTF-based HS colposcope addresses several limitations of the previous system and highlights the practical advantages of LCTF technology for HS image acquisition in clinical settings. The proposed variable exposure-time acquisition strategy demonstrated stable and consistent performance, indicating its suitability for HS systems based on spectral filtering. Together, these developments provide a robust foundation for the future development and validation of clinically relevant diagnostic support tools for the non-invasive detection and delineation of CIN lesions.

Future work will first focus on minimizing the transition artefact between filters in order to reduce the associated spectral discontinuity. In parallel, efforts will be directed toward the acquisition of larger clinical datasets, which are required for robust technical and clinical validation of machine-learning algorithms for the automated detection of precancerous cervical lesions and early-stage cervical cancer. Ultimately, integrating such algorithms into a dedicated HS colposcopy system could support more objective and reproducible clinical decision-making, thereby reducing examiner-dependent variability in cervical cancer screening and early diagnosis.

## Figures and Tables

**Table 1:** Comparative specifications and metrics of the LCTF-based and Snapscan-based HS colposºcope systems.

	LCTF System	Snapscan System
Spatial Resolution	1600 × 1600 pixels	900 × 1000 pixels
Spectral Range	460–1000 nm	470–900 nm
Number of Bands	109	158
Average FWHM	18 nm	7 nm
Maximum DR	43 dB	34 dB
Maximum SNR	43 dB (530–680 nm)	46 dB (575 nm)

## Data Availability

The datasets generated during the characterization for the current study are available from the corresponding author, under reasonable request, through https://hsidatabase.iuma.ulpgc.es/ (accessed on 15 December 2024).

## Abbreviations

The following abbreviations are used in this manuscript:

HS	Hyperspectral
HSI	Hyperspectral Imaging
LCTF	Liquid crystal tunable filters
NIR	Near-Infrared
VNIR	Visible and Near-Infrared
CIN	Cervical Intraepithelial Neoplasia
RFS	Reception-Filtering Setup
EFS	Emission-Filtering Setup
OECF	Opto-Electronic Conversion Function
RMSE	Root-Mean-Square Error
DR	Dynamic Range
SNR	Signal-to-Noise Ratio
SD	Standard Deviation
DC	Dark Current
FWHM	Full Width at Half Maximum

## References

[1] Bray F., Ferlay J., Soerjomataram I., Siegel R.L., Torre L.A., Jemal A. (2018). Global Cancer Statistics 2018: GLOBOCAN Estimates of Incidence and Mortality Worldwide for 36 Cancers in 185 Countries. CA Cancer J. Clin..

[2] Sung H., Ferlay J., Siegel R.L., Laversanne M., Soerjomataram I., Jemal A., Bray F. (2021). Global Cancer Statistics 2020: GLOBOCAN Estimates of Incidence and Mortality Worldwide for 36 Cancers in 185 Countries. CA Cancer J. Clin..

[3] Herrera A., Rojas M.D., Gabás C., de Miguel E., Ramos M., Velázquez O., Ameijide A., Galcerán J. (2018). Estimaciones de La Incidencia de Cáncer En Canarias 2018. https://www3.gobiernodecanarias.org/sanidad/scs/content/8e1d1c9c-43fd-11e9-af3a-bd8e6246c9be/Estimacion_Incidencia_Cancer_Canarias2018.pdf.

[4] Lycke K.D., Kalpathy-Cramer J., Jeronimo J., de Sanjose S., Egemen D., del Pino M., Marcus J., Schiffman M., Hammer A. (2024). Agreement on Lesion Presence and Location at Colposcopy. J. Low. Genit. Tract Dis..

[5] Brown B.H., Tidy J.A. (2019). The Diagnostic Accuracy of Colposcopy—A Review of Research Methodology and Impact on the Outcomes of Quality Assurance. Eur. J. Obstet. Gynecol. Reprod. Biol..

[6] Mustafa R.A., Santesso N., Khatib R., Mustafa A.A., Wiercioch W., Kehar R., Gandhi S., Chen Y., Cheung A., Hopkins J. (2016). Systematic Reviews and Meta-Analyses of the Accuracy of HPV Tests, Visual Inspection with Acetic Acid, Cytology, and Colposcopy. Int. J. Gynecol. Obstet..

[7] Guido R., Schiffman M., Solomon D., Burke L. (2003). Postcolposcopy Management Strategies for Women Referred with Low-Grade Squamous Intraepithelial Lesions or Human Papillomavirus DNA-Positive Atypical Squamous Cells of Undetermined Significance: A Two-Year Prospective Study. Am. J. Obstet. Gynecol..

[8] Lu G., Fei B. (2014). Medical Hyperspectral Imaging: A Review. J. Biomed. Opt..

[9] Hren R., Dóczi T., Orszagh E., Babič D. (2025). Recent Advances in Perfusion Assessment in Clinical Oncology Using Hyperspectral Imaging. Electronics.

[10] Wang C., Zheng W., Bu Y., Chang S., Zhang S., Xu R.X. (2016). Multi-Scale Hyperspectral Imaging of Cervical Neoplasia. Arch. Gynecol. Obstet..

[11] Zheng W., Wang C., Chang S., Zhang S., Xu R.X. (2015). Hyperspectral Wide Gap Second Derivative Analysis for in Vivo Detection of Cervical Intraepithelial Neoplasia. J. Biomed. Opt..

[12] Jurjuţ O., Weiss M., Daniel Y., Matovina S., Neis F., Rall K., Schöpp K., Henes M., Linzenbold W., Brucker S.Y. (2025). Detection of Cervical Intraepithelial Neoplasia Using Hyperspectral Tissue Signatures. IEEE J. Transl. Eng. Health Med..

[13] Schimunek L., Schöpp K., Wagner M., Brucker S.Y., Andress J., Weiss M. (2023). Hyperspectral Imaging as a New Diagnostic Tool for Cervical Intraepithelial Neoplasia. Arch. Gynecol. Obstet..

[14] Kamruzzaman M., Sun D.W. (2016). Introduction to Hyperspectral Imaging Technology. Computer Vision Technology for Food Quality Evaluation.

[15] SNAPSCAN VNIR Hyperspectral Camera|Imec. https://www.imechyperspectral.com/en/cameras/snapscan-vnir.

[16] Zhou X., Ma L., Mubarak H.K., Palsgrove D., Sumer B.D., Chen A.Y., Fei B. (2024). Polarized Hyperspectral Microscopic Imaging System for Enhancing the Visualization of Collagen Fibers and Head and Neck Squamous Cell Carcinoma. J. Biomed. Opt..

[17] Giannantonio T., Alperovich A., Semeraro P., Atzori M., Zhang X., Hauger C., Freytag A., Luthman S., Vandebriel R., Jayapala M., Alfano R.R., Seddon A.B. (2023). Intra-Operative Brain Tumor Detection with Deep Learning-Optimized Hyperspectral Imaging. Proceedings of the Optical Biopsy XXI: Toward Real-Time Spectroscopic Imaging and Diagnosis.

[18] Vandebriel R., Luthman S., Vunckx K., Jayapala M., Charle W., Solie L., De Vleeschouwer S., Giannantonio T., Alperovich A., Zhang X., Boudoux C., Tunnell J.W. (2023). Integrating Hyperspectral Imaging in an Existing Intra-Operative Environment for Detection of Intrinsic Brain Tumors. Proceedings of the Advanced Biomedical and Clinical Diagnostic and Surgical Guidance Systems XXI.

[19] Brunner A., Schmidt V.M., Zelger B., Woess C., Arora R., Zelger P., Huck C.W., Pallua J. (2022). Visible and Near-Infrared Hyperspectral Imaging (HSI) Can Reliably Quantify CD3 and CD45 Positive Inflammatory Cells in Myocarditis: Pilot Study on Formalin-Fixed Paraffin-Embedded Specimens from Myocard Obtained during Autopsy. Spectrochim. Acta A Mol. Biomol. Spectrosc..

[20] Yoon J. (2022). Hyperspectral Imaging for Clinical Applications. Biochip J..

[21] Zuzak K.J., Francis R.P., Wehner E.F., Litorja M., Cadeddu J.A., Livingston E.H. (2011). Active DLP Hyperspectral Illumination: A Noninvasive, in Vivo, System Characterization Visualizing Tissue Oxygenation at Near Video Rates. Anal. Chem..

[22] Wang X., Zhang Y., Ma X., Xu T., Arce G.R. (2018). Compressive Spectral Imaging System Based on Liquid Crystal Tunable Filter. Opt. Express.

[23] Clancy N.T., Jones G., Maier-Hein L., Elson D.S., Stoyanov D. (2020). Surgical Spectral Imaging. Med. Image Anal..

[24] Sahli S., Hayward J., Fang Q., Abdlaty R.M.Y.M. (2018). Hyperspectral Imaging: Comparison of Acousto-Optic and Liquid Crystal Tunable Filters. Medical Imaging 2018: Physics of Medical Imaging.

[25] Zuzak K.J., Francis R.P., Wehner E.F., Smith J., Litorja M., Allen D.W., Tracy C., Cadeddu J., Livingston E. (2009). Hyperspectral Imaging Utilizing LCTF and DLP Technology for Surgical and Clinical Applications. Proceedings of the Design and Quality for Biomedical Technologies II.

[26] Gat N., Szu H.H., Vetterli M., Campbell W.J., Buss J.R. (2000). Imaging Spectroscopy Using Tunable Filters: A Review. Wavelet Applications VII.

[27] Vega C., Medina N., Quintana-Quintana L., Leon R., Fabelo H., Rial J., Martín A., Callico G.M. (2025). Feasibility Study of Hyperspectral Colposcopy as a Novel Tool for Detecting Precancerous Cervical Lesions. Sci. Rep..

[28] (2009). Photography–Electronic Still-Picture Cameras–Methods for Measuring Opto-Electronic Conversion Functions (OECFs).

[29] Vega C., Medina N., Leon R., Fabelo H., Martín A., M. Callico G. (2026). HyCervix [Data Set]. Universidad de Las Palmas de Gran Canaria. https://doi.org/10.5281/zenodo.18208664.

[30] Vega C., Marquez-Suarez N., Leon R., Castro-Fernandez M., Socorro-Marrero G.V., Fabelo H., Rial-Ferrario J., Callico G.M. (2024). Development and Characterization of a Hyperspectral LCTF-Based Colposcopic System. Proceedings of the Liquid Crystals Optics and Photonic Devices.

[31] Mazdeyasna S., Arefin M.S., Fales A., Leavesley S.J., Pfefer T.J., Wang Q. (2025). Evaluating Normalization Methods for Robust Spectral Performance Assessments of Hyperspectral Imaging Cameras. Biosensors.

[32] Jacques S.L. (2013). Optical Properties of Biological Tissues: A Review. Phys. Med. Biol..

